# Characterization of an Ancient Lepidopteran Lateral Gene Transfer

**DOI:** 10.1371/journal.pone.0059262

**Published:** 2013-03-22

**Authors:** David Wheeler, Amanda J. Redding, John H. Werren

**Affiliations:** Department of Biology, University of Rochester, Rochester, New York, United States of America; International Atomic Energy Agency, Austria

## Abstract

Bacteria to eukaryote lateral gene transfers (LGT) are an important potential source of material for the evolution of novel genetic traits. The explosion in the number of newly sequenced genomes provides opportunities to identify and characterize examples of these lateral gene transfer events, and to assess their role in the evolution of new genes. In this paper, we describe an ancient lepidopteran LGT of a glycosyl hydrolase family 31 gene (GH31) from an *Enterococcus* bacteria. PCR amplification between the LGT and a flanking insect gene confirmed that the GH31 was integrated into the *Bombyx mori* genome and was not a result of an assembly error. Database searches in combination with degenerate PCR on a panel of 7 lepidopteran families confirmed that the GH31 LGT event occurred deep within the Order approximately 65–145 million years ago. The most basal species in which the LGT was found is *Plutella xylostella* (superfamily: Yponomeutoidea). Array data from *Bombyx mori* shows that GH31 is expressed, and low dN/dS ratios indicates the LGT coding sequence is under strong stabilizing selection. These findings provide further support for the proposition that bacterial LGTs are relatively common in insects and likely to be an underappreciated source of adaptive genetic material.

## Introduction

New coding genes can arise in genomes through several processes, including gene duplication, gene fusion, *de novo* formation from non-coding DNA, or lateral gene transfer (LGT) from another species. In bacteria, frequent LGT events play a major role in the plasticity of genomes and contribute to their characteristic adaptability [1]. In contrast, LGTs were previously thought to play only a minor role in the evolution of animal genomes, based on the paucity of known examples and the requirement that heritable changes must enter the germline. However, recent research, mostly in insects and nematodes, has highlighted that bacterial to animal LGTs may in fact be a much more important source of novel gene evolution than was originally thought [2], [3].

Once an LGT has occurred, it will either degrade through mutational processes (deletions and mutations) or, in some cases, evolve into a functional gene. The latter process is poorly understood, but several examples have been found, including actively transcribed *Wolbachia* LGTs in the parasitoid *Nasonia vitripennis* [4], fungal-origin genes for carotenoids in aphids and spider mites [5], [6], and bacterially derived plant cellulases in plant parasitic nematodes [7]. These and many other examples of LGTs can contribute to the adaptive potential of the recipient. This latter point was particularly well highlighted in a recent report that a mannanase gene in the genome of the coffee borer beetle (*Hypothenemus hampei*) is result of an LGT with a *Bacillus* bacterium [8]. Acuña et al. (2012) [8] posit that the *H. hampei* LGT may be responsible for processing galactomannan, which is the most common polysaccharide in coffee beans, thus facilitating specialization of this pest species on coffee plants.

The insects have attracted much attention in the search for microbial LGTs, partially because many are infected by the endosymbiotic alpha-proteobacteria *Wolbachia*, whose close association with the germline provides opportunities for heritable cross-kingdom transfer of DNA. Putative LGTs involving *Wolbachia* have also been found in wasps, mosquitoes, aphids, beetles, filarial nematodes, and tsetse flies [2], [4], [9]–[14]. Other insect associated bacteria have also been donors in LGT events, with these transfers likely mediated through occupation of the bacteria in the gastrointestinal and reproductive tracts, as well as parasitic and/or intracellular life stages [15]–[17]. Recently, Li et al. (2011) [18] carried out a screen of LGTs in several insect species with a bioinformatic pipeline. Somewhat surprisingly, LGTs were only detected in *B. mori*, with a broad range of donor species encompassing 11 genera. A complicating factor in searches for LGTs is that many genomes contain contaminating DNA sequences from bacteria that were associated with the sequenced animal. Highlighting this problem Li et al. (2011) [18] identified 77 genes on contaminating bacterial scaffolds that remain part of the Official Gene Set release 2 for *Apis mellifera*. This latter point illustrates the importance of confirming the localization of the LGT in animal genomes, either by PCR and sequencing over insect bacteria boundaries or by *in situ* hybridization.

The rapid expansion in newly sequenced genomes provides opportunities to identify additional examples of LGT events between bacteria and animals. In this study we make use of the recently completed *Danaus plexippus* genome to identify a bacterial LGT of a glycosyl hydrolase from *Enterococcus,* show that the LGT is ancient in Lepidoptera, and for one species provide molecular evidence for its insertion into the genome. The high conservation of the protein coding sequence for this LGT among Lepidoptera that have diverged over 65–145 million years ago (MYA) and evidence of its expression, provides strong support that this gene is functional.

## Materials and Methods

### Bioinformatics

The *D. plexippus* genomic scaffolds and gene model information (OGS1) were downloaded from MonarchBase (http://monarchbase.umassmed.edu/home.html). To identity potential recent LGT events we used BLASTN to compare the *D. plexippus* genomic scaffolds against a bacterial database containing 1,097 complete bacterial genome sequences downloaded from the National Center for Biotechnology Information (NCBI). Regions with significant bacterial identity (E value<1e^−5^) were then compared to a second database containing representative animal genomes (*Homo sapiens, Mus musculus, Rattus rattus, Monodelphis domestica, Gallus gallus, Xenopus laevis, Drosophila melanogaster, Anopheles gambiae, N. vitripennis, A. mellifera, Daphnia magna*) to obtain a corresponding “animal” BLASTN E value score. If the animal E value score was less than the original E value score the sequence was excluded as a slowly evolving highly conserved gene. As smaller genomic scaffolds tend to be over-represented by DNA from environmental sources (DW unpublished observation) we also conservatively excluded any candidate region that was located in a scaffold smaller than 5 Kb in length. One to one BLAST orthologs were identified by reciprocal BLASTP searches between *D. plexippus* and the *B. mori* OGS peptides (downloaded from SilkDB http://silkworm.genomics.org.cn/silkdb). The *P. xylostella* OGS peptides were downloaded from the diamondback moth genome database (http:://iae.fafu.edu.cn/DBM).

### Evolutionary Techniques

Non- synonymous to synonymous (dNdS) ratios were derived from a DNA alignment of the BmGH31, DpGH31, and PxGH13 coding regions using PAML [19]. The parameters used in the calculations of dNdS were runmode = −2, allow unequal codon frequencies (CodonFreq = 2) and estimate transition to transversion rate ratio (fix_kappa = 0). For Bayesian phylogenetic analysis putative homologs of the DpGH31 were identified using BLASTP searches of the NR database at NCBI. Sequences were aligned using CLUSTALW (www.ebi.ac.uk/Tools/msa/clustalw2) using the default options. The resulting CLUSTALW alignment was manually trimmed to include the GH31 super family domain (Conserved domain database: cl11402) that contained the strongest homology amongst these divergent sequences. Phylogenetics was performed using MrBayes (version 3.2.1) [20] with four independent runs of 3 million generations with sampling every 1000 generations and 25% of samples discarded as a ‘burnin’. The models of sequence evolution used for the trees where were WAG+I+G (Evolution of Prokaryote and Eukaroyote GH31 sequences), WAG+G (bacterial source of LGT), and CpREV+I+G (Lepidopteran GH31 orthologs), as selected by PROTEST (version 2.4) [21]. Branching arrangements of parsimony trees estimated using PHYLIP (version 3.69) [22] were the same as those in the Bayesian tree at nodes with strong bootstrap support (>80% Bootstrap values based on 1000 pseudoreplicates of the data). To identify possible sources of the lepidopteran LGT we downloaded the pre-compiled bacterial protein databases from PATRIC (www.patricbrc.org) and used BLASTP with the BmGH31 and DpGH31 protein sequences to identify homologs. To reduce the size of the alignment we filtered candidates based on a maximum of 5 representatives per species. A protein alignment, generated using CLUSTALW, was then used to derive a Bayesian tree in MrBayes using the run parameters described above.

### Degenerate PCR and Sequencing

The degenerate primers DM1 (5′TTTGGRGGNGGNATGCARAAYGG3′) and DM2 (5′CCRTCRTTNGGDATRAACCA3′) were designed to bind to highly conserved regions found in an alignment between DpGH31 and BmGH31 sequences. PCR conditions were 1×PCR reaction buffer, 0.4 uM each primer, 0.2 mM dNTPs, 2.0 mM MgCl_2_ and 1 unit of Taq polymerase (Invitrogen). The species tested were *Ceratomia catalpae* (Hesperiidae), *Atrytonopsis edwardsi* (Hesperiidae), *Saturnia pyri* (Saturniidae), *Grammia geneura* (Arctiidae), *Heteranassa fraternal* (Noctuidae), *Lesmone griseipennis* (Noctuidae), *Chloraspilates bicoloraria* (Geometridae), *Narraga fimetaria* (Geometridae), *Tornos erectarius* (Geometridae), *Eurema lisa* (Pieridae), *Euptoieta claudia* (Nymphalidae), *Gonometa rufobrunnea* (Lasiocampidae), *Poecilacampa populi* (Lasiocampidae), *Argyrotaenia velutinana* (Tortricidae) and *Lymantria dispar* (Lymantriidae). Despite several attempts we were unable to amplify a PCR product from *L. dispar* genomic DNA using DM1/DM2. The identity of all successful PCR products was confirmed using Sanger sequencing and the sequences have been deposited in GenBank under accessions: KC577819-KC577832. PCR primers used to amplify between BmGH31 and the neighboring gene (BGIBMGA013897) on scaffold 3099 were DW72 (5′ CATTCAACGATCTGAAATGCC 3′) and DW73 (5′ CGCGGAGATCCGGATGGTC 3′).

## Results

### Bioinformatic Identification of a *D. plexippus* LGT

To identify relatively recent LGT regions in the *D. plexippus* genome we used BLASTN searches of the genomic scaffolds against a database of taxonomically diverse bacterial genomes (see methods). In contrast to other studies [18], [23], we decided to use a BLASTN based approach that utilizes the rapid decay of DNA homology to greatly reduces the likelihood of false positives resulting from functionally constrained eukaryotic protein motifs that are shared by bacteria. The single best candidate LGT in the *D. plexippus* genome based on the BLASTN searches, with a strong BLAST match to the glycosyl hydrolase family 31 (GH31) from *Enterococcus faecalis*, was on scaffold 15050 located within a predicted gene called *DPGLEAN20412* (henceforth referred to as DpGH31). Protein BLAST searches using the DpGH31 sequence against the NCBI NR database also returned a best hit to a glycosyl hydrolase of *E. faecalis* strain TX0104 (56% identity; E value of 0.0). We noted that the BLASTP output for the gene also contained a strong hit (E value of 0.0) to a glycosyl hydrolase (*BGIBMGA013995*) from the genome of *B. mori*. A previous report has described *BGIBMGA013995* as a possible LGT [18]. Reciprocal BLASTP comparisons of all *D. plexippus* and *B. mori* proteins revealed that DpGH31 and BGIBMGA013995 (henceforth referred to as BmGH31) are 1∶1 BLAST orthologs. The LGT was identified in *Plutella xylostella* by performing a BLASTP search with the BmGH31 peptide sequence against the *P. xylostella* peptide database, available at the diamondback moth genome database (http://iae.fafu.edu.cn/DBM) [24]. The top hit (E value 0.0) was to Px016165, henceforth referred to as PxGH31. The best animal BLASTP hit to DpGH31 is alpha glucosidase II alpha subunit from *Hydra magnipapillata* (E value 7e^−40^) and the best insect hit (E value 1e^−35^) is alpha-glucosidase AB from the ant *Camponotus floridanus*. These BLAST results support the conclusion that DpGH31 represents an LGT between an *Enterococcus* bacteria and the common ancestor of *D. plexippus*, *B. mori,* and *P. xylostella* (a conclusion corroborated by phylogenetic evidence below).

### Gene Structure and Expression of the GH31 LGT

**Figure 1:**
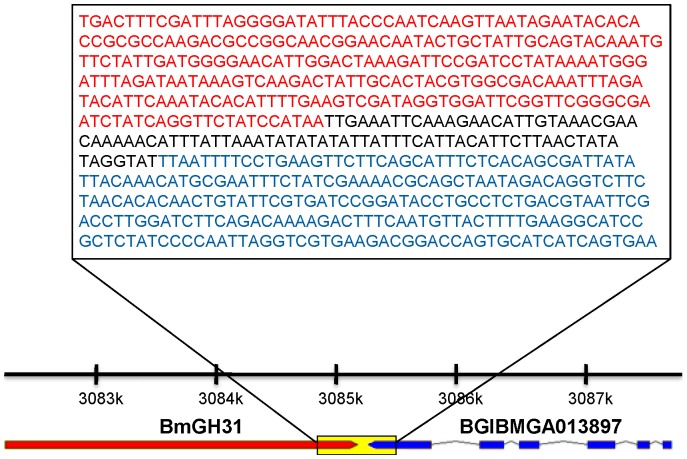
Molecular evidence supports that BmGH31 is part of the *B. mori* genome. PCR was used to amplify across the boundary between the BmGH31 LGT and a flanking insect gene called *BGIBMGA013897* that is conserved in divergent insect species. The sequence boundary between BmGH31 (red) and *BGIBMGA013897* (blue) was obtained by sequencing the PCR product. The scaffold positions along scaffold3899 are shown above the gene models with the region amplified by the PCR is highlighted by the box. Note that the coding region of BGIBMGA013897 is located on the opposite strand.

**Figure 2:**
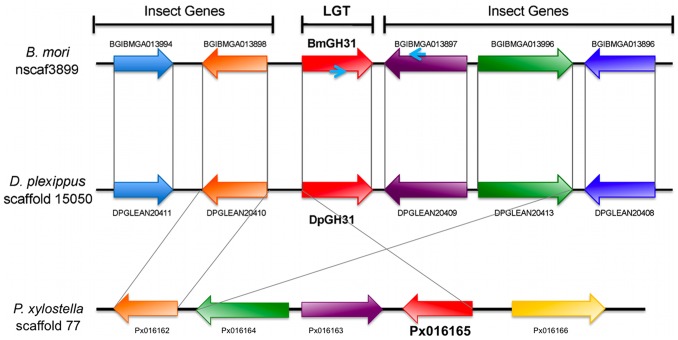
Microsynteny exists in the region surrounding DpGH31, BmGH31 and PxGH31. Microsynteny of genes surrounding the GH31 LGT regions in *B. mori*, *D. plexippus* and *P. xylostella*. The genes’ direction of transcription is indicated by arrows with LGTs shown in red. Genes that are one to one blast orthologs between *B. mori, D. plexippus,* and *P. xylostella* are indicated by matching colors and connecting gray lines. The PCR primers used to amplify between BmGH31 and the neighboring gene (BGIBMGA013897) on scaffold 3099 are indicated by small blue arrows on BmGH31 and BGIBMGA013897.

Consensus gene models for both BmGH31 and DpGH31 predict intronless genes that encode 1076 and 923 amino acid long peptide sequences, respectively. A single AUGUSTUS gene model for DpGH31 predicts a small 24 bp intron at the 3′ end of the gene, this exonic/intronic arrangement seems unlikely given it is not independently supported by protein alignments to other GH31 sequences. Eukaryotic gene features such as near consensus poly-A attachment site could be identified in both putative LGTs, whilst neither sequence contained Shine Delgarno sequences that are typically required for bacterial gene translation [25] (results not shown). The pairwise amino acid identity (58.5%) between BmGH31 and DpGH31 was not significantly different from the genome average (70.7%) for other *B. mori* and *D. plexippus* 1 to 1 orthologs (Z score = −0.78; p>0.05). Furthermore, DpGH31 and BmGH31 show a very low non-synonymous to synonymous substitution ratio (dN/dS = 0.062), indicative of actively transcribed protein coding genes under strong purifying selection. Both also show low dN/dS to PxGH31 in the more distantly related *Plutella xylostella* (dN/dS = 0.107 to Dp and 0.108 to Bm). Finally, to rule out the possibility that BmGH31 represents a bacterial insect chimeric miss assembly, we designed PCR primers that would amplify between the LGT and a neighboring gene within scaffold 3099 (Fig. 1). Based on the annotation of *B. mori* scaffold 3099, BmGH31 is located 85 bp from a *BGIBMGA013897,* which encodes a novel protein conserved across several insect and animal orders (thus representing a “typical” insect gene). PCR using *B. mori* genomic DNA with the bridging primers successfully amplifying the expected product size and sequencing confirmed that BmGH31 is located within the *B. mori* genome (Fig. 1). Although, we did not directly confirm the scaffold arrangement for *D. plexippus* and *P. xylostella* by PCR, the preservation of microsynteny between independent assemblies of the DpGH31 genomic region, the PxGH31 genomic region, and the BmGH31 genomic region strongly supports the conclusion that DpGH31 and PxGH31 are also part of the Monarch and Diamondback moth genomes, respectively (Fig. 2).

Microarray data indicates that BmGH31 is expressed in the testis, with moderate to low expression in the ovary, malpighian tubules, fat body, and integument [18]. We were unable to identify an EST for DpGH31 in either the NCBI EST or transcriptome shotgun assembly databases, though it should be noted these are largely brain-specific libraries and MonarchBase indicates that 1635 RNAseq reads map to this gene. Given the above observations plus absence of other evidence that would suggest these genes are pseudogenes (i.e. stop codons, elevated dNdS), it is highly likely that both genes are functionally important.

### Phylogenetic Relationships of GH31

**Figure 3:**
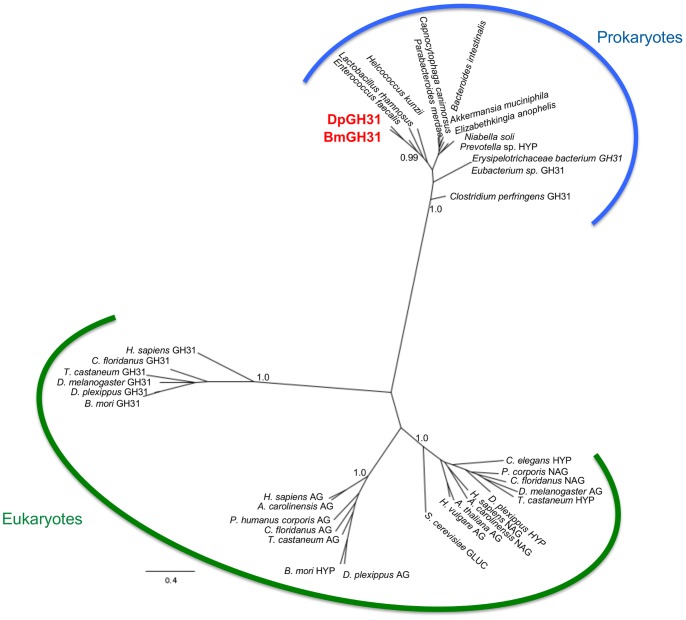
Bayesian phylogeny of DpGH31 and BmGH31 sequences. Unrooted Bayesian phylogenetic tree of GH31-like protein sequences from prokaryote and eukaryote species. The clade grouping the *D. Plexippus* and *B. mori* LGTs (DpGH31 and BmGH31 shown in red) with the *E. faecalis* GH31 sequence is supported by a 100% posterior probability. The division of the sequences into Prokaryote and Eukaryote clades, indicted by the curved lines, is supported by a >98% posterior probabilities, with the Lepidopteran LGTs clearly within the bacterial clade. Only posterior probability values at key nodes are not shown for clarity. The tentative protein annotations were made based on the information available at NCBI using the following key: hypothetical (HYP), glycosyl hydrolase family 31 (GH31), alpha-glucosidase (AG), neutral alpha-glucosidase (NAG), glucosidase (GLUC). Gene identifier numbers for all sequences in the phylogeny are provided in Table S1.

To better understand the evolutionary history of the *D. plexippus* and *B. mori* GH31 LGT sequences, we carried out a phylogenetic analyses with homologous sequences from eukaryote and prokaryote species. In concordance with the BLAST results the Bayesian phylogenetic tree (Fig. 3) groups DpGH31 and BmGH31 together within a strongly supported clade (Posterior probability 99%) of bacterial GH31 sequences. The three other well supported clades (Posterior probability 100%) are composed of animal GH31-like proteins, animal alpha-glucosidases and eukaryotic neutral alpha glucosidase sequences (Fig. 3). Importantly, typical insect alpha-glucosidases found in the genomes of both *B. mori* and *D. plexippus* are placed into the expected insect clades. Therefore, the phylogeny strongly suggests DpGH31 and BmGH31 result from an LGT between a bacteria and the common ancestor of *B. mori* and *D. plexippus* at least 65 MYA [26].

### *E. faecalis* is the Most Likely Source of the GH31 LGT

**Figure 4:**
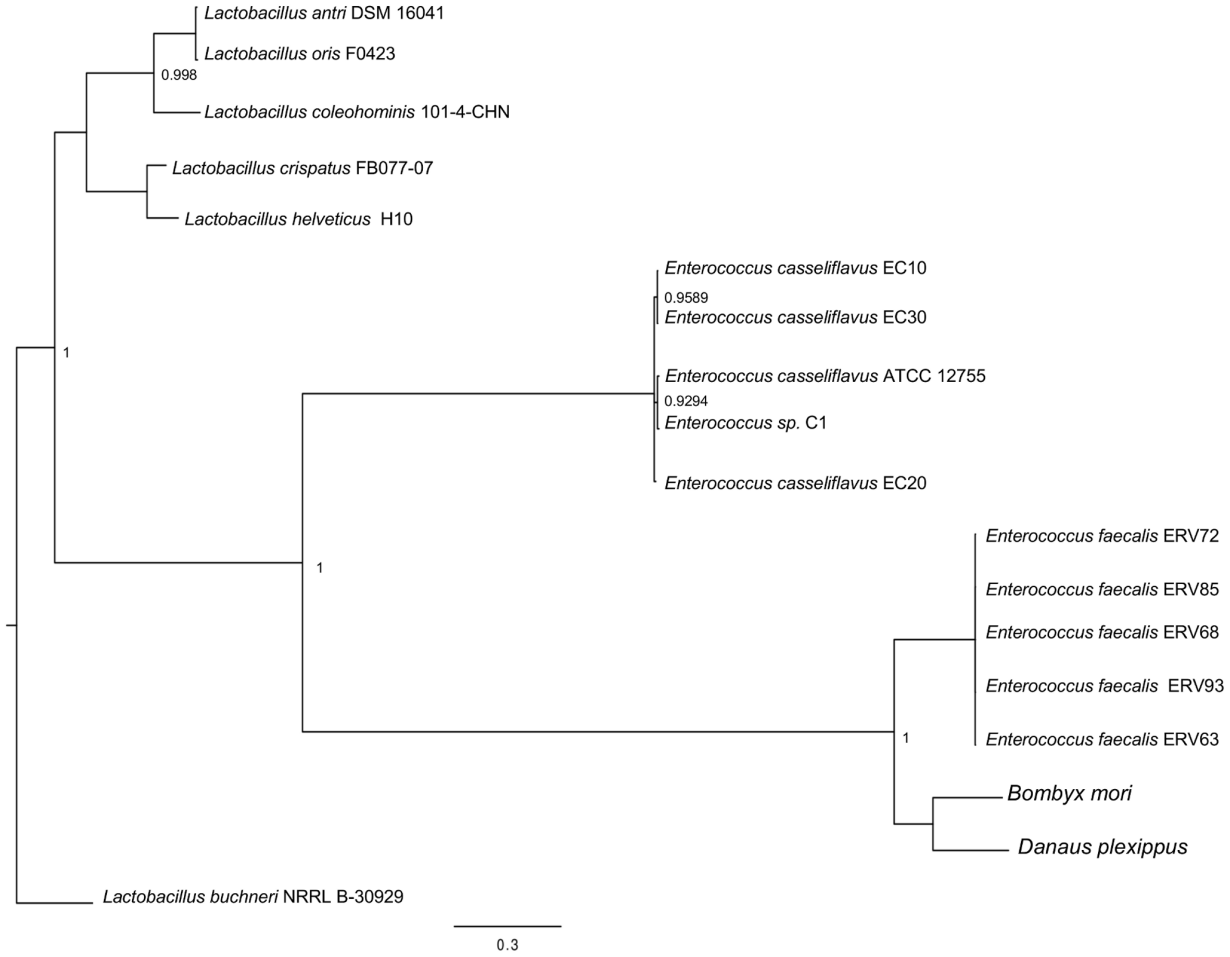
*E. faecalis* is the likely source of LGT. Bacilli homologs of DpGH31 and BmGH31 were identified using BLASTP searches of a whole genome microbial databases available at Pathosystems Integration Resource Center (PATRIC). The resulting protein sequences were used to generate the Bayesian phylogeny that shows DpGH31 and BmGH31 grouping (100% posterior probability) with *E. faecalis* strains.

To better understand the source of the LGT, we used BLASTP searches to identify homologs of DpGH31 and BmGH31 from the Class Bacilli of complete bacteria genomes. The resulting phylogeny constructed from these GH31 sequences (Fig. 4) strongly supports (Posterior probability 100%) *E. faecalis* (or a closely related, unsequenced *Enterococcus*) as the source of DpGH31 and BmGH31, from a single event in the common ancestor of *B. mori* and *D. plexippus*.

### GH31 Orthologs in Other Lepidopteran Species

**Figure 5:**
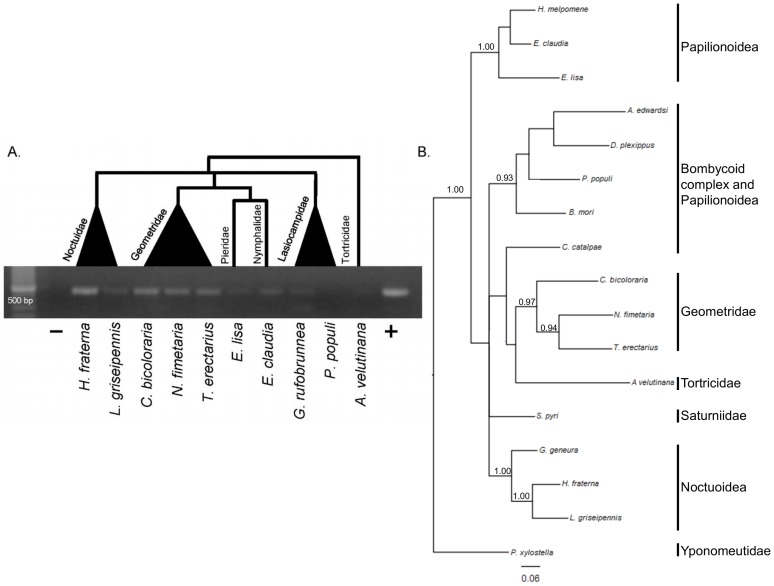
The GH31 LGT is ancient in origin. A.) Putative homologs of DpGH31 identified using degenerate PCR on genomic DNA from a panel of diverse lepidoptera species. Phylogenetic relationships of the families are taken from Cho et al 2011 [36]. *Bombxy mori* genomic DNA was used as the positive control (+). Faint bands could be observed for *P. populi* and *A. velutinana* on the original gel. B.) A Bayesian phylogenetic tree derived from GH31-LGT conceptually translated DNA sequences. DNA sequences used were obtained either by degenerate PCR or searches of available lepidopteran genome sequences. Well supported clades are resolved for members of the Noctuoidea and Geometroidea. The phylogenetic relationships of species from the Bombycoid complex and the butterflies could not be resolved in this analysis (posterior probability values less than 90%).

**Figure 6:**
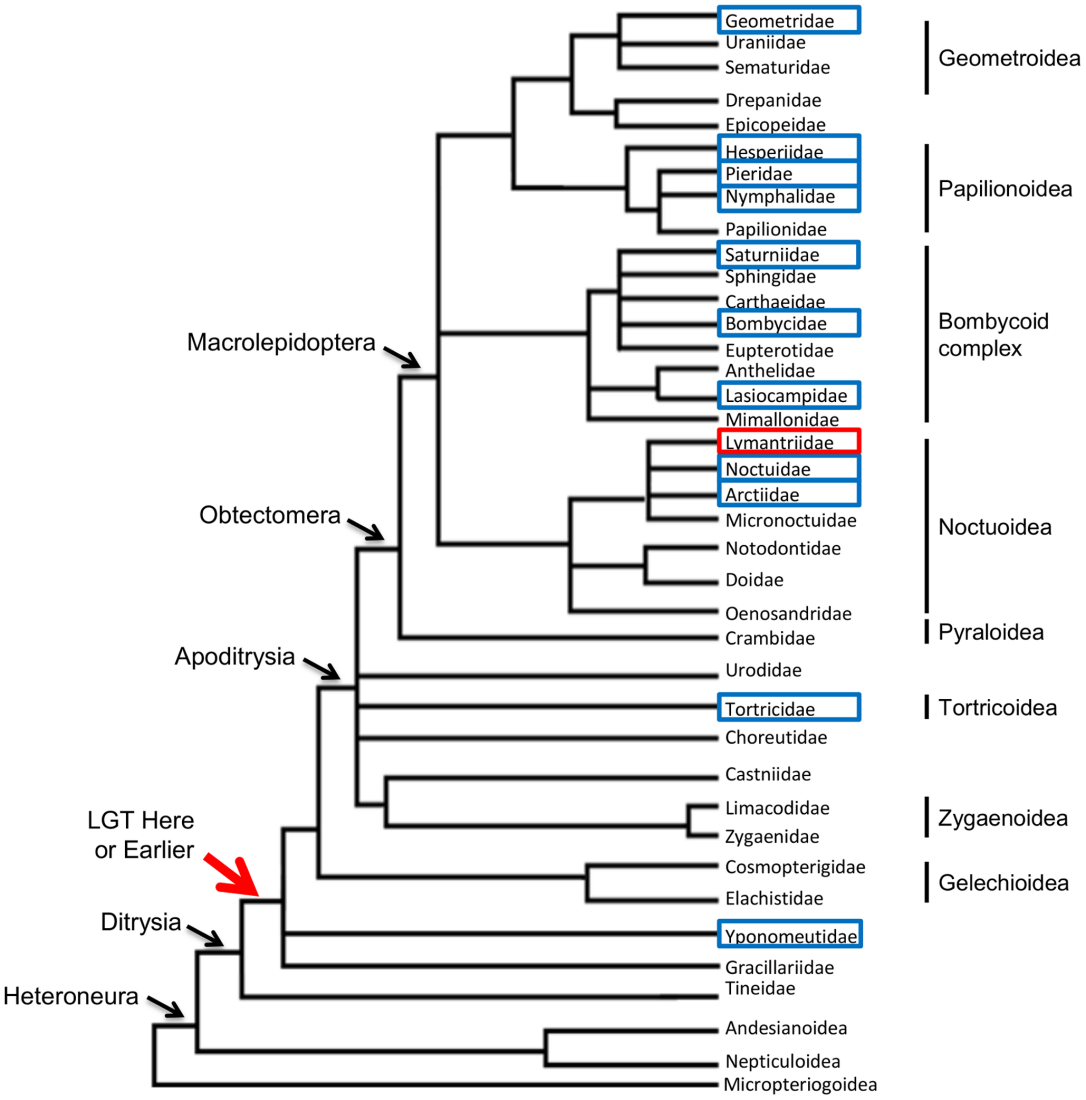
A branching diagram showing the hypothesized relationships of lepidopteran families. PCR with sequencing, and bioinformatic searches were used to identify GH31 LGTs in the lepidopteran species from families highlighted by the blue boxes. The only family in which the LGT was not confirmed by degenerate PCR is Lymantriidae (red box). The most basal species in which the LGT was discovered is *P. xylostella* (Yponomeutidae). This places the timescale of LGT acquisition (red arrow) to the Cretaceous (145-66 mya), as this is when early radiations of ditrysian superfamilies likely occurred [26]. The branch arrangements are based on data from [28], [36], [37].

Based on bioinformatic searches, Li et al. (2011) [18] previously reported that BmGH31 was limited to the Bombycoidea. The identification of DpGH31 in *D. plexippus* (Nymphalidae) motivated us to test several lepidopteran super families for the presence of homologous LGTs. To do this we used conserved regions in the DpGH31 and BmGH31 protein sequences to design degenerate primers that could be used for PCR screens. For this PCR assay we screened species from the Noctuidae, Geometridae, Pieridae, Nymphalidae, Lasiocampidae, Lymantriidae, and Tortricidae. The degenerate primers successfully amplified products with the predicted size from all these taxonomically and geographically diverse groups of lepidopteran species (Fig. 5A), except for the gypsy moth (Lymantriidae; results not shown). The product for *P. populi* and *A. velutina* amplified poorly even after attempts to optimize the PCR, suggesting that the degenerate primers are not universally efficient across the Lepidoptera. We also were able to bioinformatically identify a GH31-like gene called *HMEL002892* with strong BLAST similarity to DpGH31 in the recently published genome of *Heliconius melpomene*. Conservation of microsynteny between *HMEL002892,* DpGH31, and BmGH31 provides further support that this *H. melpomene* gene is also related to the LGT event (Fig. 2; results not shown). Although the super family relationships of Lepidoptera are currently unresolved (see Kim et al. (2011) [27]), a phylogeny construction using the lepidopteran inferred amino acid sequences from the sequenced PCR-amplified products from species in Figure 5B generally agrees with the established phylogeny at the family level [28], supporting the orthologous relationship of these sequences. Well-supported clades are found for members of the Noctuoidea and Geometroidea, while other phylogenetic relationships were not resolved. For example, *S. pyri* does not group with the other bombycoid complex species, this most likely results from the relatively few phylogenetic informative sites found in the small region used to derive the phylogeny (116 amino acids). The results demonstrate that DpGH31 homologs are widespread within Lepidoptera. The LGT event occurred around or before the diversification of the Ditrysia (Fig. 6), which has been dated to the Cretaceous period. These findings therefore suggest that the LGT event occurred between 145-65 MYA [26], [29]. At this stage the failure to amplify a product in the gypsy moth (family Lymantriidae) requires further investigation before it can be concluded that this species lost the LGT.

### Possible Functions of Lepidopteran GH31 LGT

The DpGH31 sequence contains three protein family domains: glycoside hydrolase family 31 (PF01055), Discoidin (PF00754), and a Fibronectin type III (PF00041). These domains are extremely common in nature and are involved in a diverse array of mechanisms, including primary metabolism, glycosylation, and immunity. The GO annotation of BmGH31 is a hydrolysis of O-glycosyl compounds is also a very general functional category.

## Discussion

In this study we used bioinformatic, molecular and phylogenetic methods to characterize an ancient LGT between an *Enterococcus* and an ancestral lepidopteran 145-65 MYA. The most basal lineage that the LGT was found in is *P. xylostella* (superfamily Yponomeutoidea). We have not yet resolved whether the LGT is present in Tineoidea (which includes bagworms [30]), so the LGT could be even more ancient in the Lepidoptera. The use of molecular methods to confirm that BmGH31 is part of the *B. mori* genome, together with the conservation of microsynteny in this region with *D. plexippus* and *P. xylostella*, makes it highly unlikely that these genes are based on a chimeric assembly between bacterial and insect DNA (Fig. 1). Conservation of the LGT protein coding sequence (relative to synonymous substitutions) within several diverse lepidopteran families and its expression in *B. mori* provides strong evidence that the LGT is functional (Fig. 5).

The actual function of the GH31 LGT in Lepidoptera is currently unknown. Unfortunately, bioinformatic and evolutionary tools provide few clues to actual biological function of BmGH31 and DpGH31, as GH31 members are associated with many molecular pathways. For example, other members of this family are involved in the digestion of starch in the human intestine, however, BmGH31 is only expressed lowly in the gut of *B. mori* [23], [31]. The high expression in the gonad also provides few clues to a possible function as the literature is sparse on examples of testis specific GH31 members. In the future we hope to directly examine the function of BmGH31 using RNA interference gene knockdowns, a method that has previously been successful in *B. mori* functional studies [32]–[34]. Currently, there is no spatial or temporal expression data available for *D. plexippus*, and although we were unable to identify an EST for DpGH31, the existing EST libraries are mostly derived from brain tissue.

The mechanisms that evolve LGTs into functional eukaryotic genes are poorly understood and fascinatingly complex. The acquisition of a eukaryotic promoter could occur quickly if the LGT integrates near an existing promoter or enhancer element. However, the evolution of introns would be more complex and presumably represents a multistep process. With this latter point in mind, it is interesting that neither *D. plexippus* nor *B. mori* GH31 sequences contain introns, despite their ancient establishment. Li et al. (2011) [18] also noted that 20 other putative LGTs they identified in *B. mori* are intronless. Similarly, LGTs involving two *Wolbachia* genes in the genome of *Aedes aegypti* also are intronless [11]. The formation of eukaryotic splice sites must direct precise splicing such that the reading frame in the mRNA is preserved. The process of intron acquisition is thus likely to be hindered by the high probability of deleterious intermediate steps, such as the introduction of frame shift mutations or protein truncations. That several examples of intron-containing LGTs exist shows that this process does occur. However, with the present case in mind, it may require many millions of years [4], [10], [35]. Our limited understanding of intron function also makes it difficult to devise clear hypothesis to explain the selective pressures driving intron acquisition in LGTs. It is clear that the study of LGTs will provide us with a rich source of information not only on the evolution of new introns, but also on the selective forces driving LGT maintenance within eukaryotes.

## Supporting Information

Table S1 GI numbers for protein sequences used in the phylogenetic tree shown in Figure 3.
(DOC)

## References

[1] Ochman H, Lawrence JG, Groisman EA (2000) Lateral gene transfer and the nature of bacterial innovation. Nature 405: 299–304 doi:10.1038/35012500. 10830951 10.1038/35012500

[2] Hotopp JCD, Clark ME, Oliveira DCSG, Foster JM, Fischer P, et al (2007) Widespread Lateral Gene Transfer from Intracellular Bacteria to Multicellular Eukaryotes. Science 317: 1753–1756 doi:10.1126/science.1142490. 17761848 10.1126/science.1142490

[3] Dunning Hotopp JC (2011) Horizontal gene transfer between bacteria and animals. Trends Genet 27: 157–163 doi:10.1016/j.tig.2011.01.005. 21334091 10.1016/j.tig.2011.01.005 PMC3068243

[4] Werren JH, Richards S, Desjardins CA, Niehuis O, Gadau J, et al (2010) Functional and evolutionary insights from the genomes of three parasitoid Nasonia species. Science 327: 343–348. 20075255 10.1126/science.1178028 PMC2849982

[5] Moran NA, Jarvik T (2010) Lateral Transfer of Genes from Fungi Underlies Carotenoid Production in Aphids. Science 328: 624–627 doi:10.1126/science.1187113. 20431015 10.1126/science.1187113

[6] Altincicek B, Kovacs JL, Gerardo NM (2012) Horizontally transferred fungal carotenoid genes in the two-spotted spider mite Tetranychus urticae. Biol Lett 8: 253–257 doi:10.1098/rsbl.2011.0704. 21920958 10.1098/rsbl.2011.0704 PMC3297373

[7] Keen NT, Roberts PA (1998) Plant parasitic nematodes: Digesting a page from the microbe book. PNAS 95: 4789–4790. 9560178 10.1073/pnas.95.9.4789 PMC33851

[8] Acuña R, Padilla BE, Flórez-Ramos CP, Rubio JD, Herrera JC, et al (2012) Adaptive horizontal transfer of a bacterial gene to an invasive insect pest of coffee. PNAS 109: 4197–4202 doi:10.1073/pnas.1121190109. 22371593 10.1073/pnas.1121190109 PMC3306691

[9] McNulty SN, Foster JM, Mitreva M, Dunning Hotopp JC, Martin J, et al (2010) Endosymbiont DNA in Endobacteria-Free Filarial Nematodes Indicates Ancient Horizontal Genetic Transfer. PLoS ONE 5: e11029 doi:10.1371/journal.pone.0011029. 20543958 10.1371/journal.pone.0011029 PMC2882956

[10] Nikoh N, Nakabachi A (2009) Aphids acquired symbiotic genes via lateral gene transfer. BMC Biol 7: 12 doi:10.1186/1741-7007-7-12. 19284544 10.1186/1741-7007-7-12 PMC2662799

[11] Klasson L, Kambris Z, Cook P, Walker T, Sinkins S (2009) Horizontal gene transfer between Wolbachia and the mosquito Aedes aegypti. BMC Genomics 10: 33 doi:10.1186/1471-2164-10-33. 19154594 10.1186/1471-2164-10-33 PMC2647948

[12] Nikoh N, Tanaka K, Shibata F, Kondo N, Hizume M, et al (2008) Wolbachia genome integrated in an insect chromosome: Evolution and fate of laterally transferred endosymbiont genes. Genome Res 18: 272–280 doi:10.1101/gr.7144908. 18073380 10.1101/gr.7144908 PMC2203625

[13] Doudoumis V, Alam U, Aksoy E, Abd-Alla AMM, Tsiamis G, et al. (n.d.) Tsetse-Wolbachia symbiosis: Comes of age and has great potential for pest and disease control. J. Invertebr. Pathol. Published online ahead of print. doi: 10.1016/j.jip.2012.05.010. 10.1016/j.jip.2012.05.010 PMC3772542 22835476

[14] Doudoumis V, Tsiamis G, Wamwiri F, Brelsfoard C, Alam U, et al (2012) Detection and characterization of Wolbachia infections in laboratory and natural populations of different species of tsetse flies (genus Glossina). BMC Microbiol 12: S3 doi:10.1186/1471-2180-12-S1–S3. 22376025 10.1186/1471-2180-12-S1-S3 PMC3287514

[15] Mason KL, Stepien TA, Blum JE, Holt JF, Labbe NH, et al (2011) From Commensal to Pathogen: Translocation of Enterococcus faecalis from the Midgut to the Hemocoel of Manduca sexta. mBio 2: e00065–11 doi:10.1128/mBio.00065-11. 21586646 10.1128/mBio.00065-11 PMC3101781

[16] Tang X, Freitak D, Vogel H, Ping L, Shao Y, et al (2012) Complexity and Variability of Gut Commensal Microbiota in Polyphagous Lepidopteran Larvae. PLoS ONE 7: e36978 doi:10.1371/journal.pone.0036978. 22815679 10.1371/journal.pone.0036978 PMC3398904

[17] Martin JD, Mundt JO (1972) Enterococci in Insects. Appl Microbiol 24: 575–580. 4628796 10.1128/am.24.4.575-580.1972 PMC380616

[18] Li Z-W, Shen Y-H, Xiang Z-H, Zhang Z (2011) Pathogen-origin horizontally transferred genes contribute to the evolution of Lepidopteran insects. BMC Evol Biol 11: 356 doi:10.1186/1471-2148-11-356. 22151541 10.1186/1471-2148-11-356 PMC3252269

[19] Yang Z (2007) PAML 4: Phylogenetic Analysis by Maximum Likelihood. Mol Biol Evol 24: 1586–1591 doi:10.1093/molbev/msm088. 17483113 10.1093/molbev/msm088

[20] Ronquist F, Teslenko M, Mark P van der, Ayres DL, Darling A, et al (2012) MrBayes 3.2: Efficient Bayesian Phylogenetic Inference and Model Choice Across a Large Model Space. Syst Biol 61: 539–542 doi:10.1093/sysbio/sys029. 22357727 10.1093/sysbio/sys029 PMC3329765

[21] Abascal F, Zardoya R, Posada D (2005) ProtTest: selection of best-fit models of protein evolution. Bioinformatics 21: 2104–2105 doi:10.1093/bioinformatics/bti263. 15647292 10.1093/bioinformatics/bti263

[22] Felsenstein J (1989) PHYLIP - Phylogeny Inference Package (Version 3.2). Cladistics 5: 164–166.

[23] Zhu B, Lou M-M, Xie G-L, Zhang G-Q, Zhou X-P, et al (2011) Horizontal gene transfer in silkworm, Bombyx mori. BMC Genomics 12: 248 doi:10.1186/1471-2164-12-248. 21595916 10.1186/1471-2164-12-248 PMC3116507

[24] You M, Yue Z, He W, Yang X, Yang G, et al.. (2013) A heterozygous moth genome provides insights into herbivory and detoxification. Nat Genet. Published online ahead of print. doi:10.1038/ng.2524. 10.1038/ng.2524 23313953

[25] Shine J, Dalgarno L (1974) The 3′-Terminal Sequence of Escherichia coli 16S Ribosomal RNA: Complementarity to Nonsense Triplets and Ribosome Binding Sites. PNAS 71: 1342–1346. 4598299 10.1073/pnas.71.4.1342 PMC388224

[26] Grimaldi G, Engel M (2005) The evolution of the insects. New York: Cambridge University Press. 772 p.

[27] Kim MJ, Kang AR, Jeong HC, Kim K-G, Kim I (2011) Reconstructing intraordinal relationships in Lepidoptera using mitochondrial genome data with the description of two newly sequenced lycaenids, Spindasis takanonis and Protantigius superans (Lepidoptera: Lycaenidae). Mol Phylogenet Evol 61: 436–445 doi:10.1016/j.ympev.2011.07.013. 21816227 10.1016/j.ympev.2011.07.013

[28] Regier JC, Zwick A, Cummings MP, Kawahara AY, Cho S, et al (2009) Toward reconstructing the evolution of advanced moths and butterflies (Lepidoptera: Ditrysia): an initial molecular study. BMC Evol Biol 9: 280 doi:10.1186/1471-2148-9-280. 19954545 10.1186/1471-2148-9-280 PMC2796670

[29] Whitfield JB, Kjer KM (2008) Ancient Rapid Radiations of Insects: Challenges for Phylogenetic Analysis. Annu Rev Entomol 53: 449–472 doi:10.1146/annurev.ento.53.103106.093304. 17877448 10.1146/annurev.ento.53.103106.093304

[30] Rhainds M, Davis DR, Price PW (2009) Bionomics of Bagworms (Lepidoptera: Psychidae)*. Annu Rev Entomol 54: 209–226 doi:10.1146/annurev.ento.54.110807.090448. 18767982 10.1146/annurev.ento.54.110807.090448

[31] Eskandari R, Jones K, Ravinder Reddy K, Jayakanthan K, Chaudet M, et al (2011) Probing the Intestinal α-Glucosidase Enzyme Specificities of Starch-Digesting Maltase-Glucoamylase and Sucrase-Isomaltase: Synthesis and Inhibitory Properties of 3′- and 5′-Maltose-Extended De-O-sulfonated Ponkoranol. Chem-Eur J 17: 14817–14825 doi:10.1002/chem.201102109. 22127878 10.1002/chem.201102109

[32] Quan GX, Kanda T, Tamura T (2002) Induction of the white egg 3 mutant phenotype by injection of the double-stranded RNA of the silkworm white gene. Insect Mol Biol 11: 217–222 doi:10.1046/j.1365-2583.2002.00328.x. 12000640 10.1046/j.1365-2583.2002.00328.x

[33] Liu C, Yamamoto K, Cheng T-C, Kadono-Okuda K, Narukawa J, et al (2010) Repression of tyrosine hydroxylase is responsible for the sex-linked chocolate mutation of the silkworm, Bombyx mori. PNAS 107: 12980–12985 doi:10.1073/pnas.1001725107. 20615980 10.1073/pnas.1001725107 PMC2919899

[34] Dai H, Ma L, Wang J, Jiang R, Wang Z, et al (2008) Knockdown of ecdysis-triggering hormone gene with a binary UAS/GAL4 RNA interference system leads to lethal ecdysis deficiency in silkworm. Acta Biochimica Bioph Sin 40: 790–795 doi:10.1111/j.1745-7270.2008.00460.x. 18776991

[35] Craig JP, Bekal S, Hudson M, Domier L, Niblack T, et al (2008) Analysis of a Horizontally Transferred Pathway Involved in Vitamin B6 Biosynthesis from the Soybean Cyst Nematode Heterodera glycines. Mol Biol Evol 25: 2085–2098 doi:10.1093/molbev/msn141. 18586696 10.1093/molbev/msn141

[36] Cho S, Zwick A, Regier JC, Mitter C, Cummings MP, et al (2011) Can Deliberately Incomplete Gene Sample Augmentation Improve a Phylogeny Estimate for the Advanced Moths and Butterflies (Hexapoda: Lepidoptera)? Syst Biol 60: 782–796 doi:10.1093/sysbio/syr079. 21840842 10.1093/sysbio/syr079 PMC3193767

[37] Kristensen N, Scoble M, Karshold O (2007) Lepidoptera phylogeny and systematics: the state of inventorying moth and butterfly diversity. Zootaxa 1668: 699–747.

