# Supplementary material for: Characterization of an Ancient Lepidopteran Lateral Gene Transfer
Source: PLoS One. 2013 Mar 22;8(3):e59262. doi: 10.1371/journal.pone.0059262 (PMC3606386; doi:10.1371/journal.pone.0059262)
Supplement: Table S1 — GI numbers for protein sequences used in the phylogenetic tree shown in Figure 3. (DOC) [file pone.0059262.s001.doc]

**Table S1. GI numbers for protein sequences used in the phylogenetic tree shown in** Figure 3.

| **Identifier used in tree** | **Species/strain** | **GI number/ID** |
| --- | --- | --- |
| Enterococcus faecalis | *Enterococcus faecalis R712* | 293383384 |
| Helcococcus kunzii | *Helcococcus kunzii ATCC 51366* | 375092330 |
| Lactobacillus rhamnosus | *Lactobacillus rhamnosus HN001* | 199597613 |
| Parabacteroides merdae | *Parabacteroides merdae ATCC 43184* | 154492568 |
| Bacteroides intestinalis | *Bacteroides intestinalis DSM 17393* | 189467470 |
| Akkermansia muciniphila | *Akkermansia muciniphila ATCC BAA-835* | 187735503 |
| Prevotella sp. HYP | *Prevotella sp. oral taxon 302 str. F0323* | 357060497 |
| Elizabethkingia anophelis | *Elizabethkingia anophelis Ag1* | 365874790 |
| Capnocytophaga canimorsus | *Capnocytophaga canimorsus Cc5* | 340622823 |
| Clostridium perfringens GH31 | *Clostridium perfringens D str. JGS1721* | 182626206 |
| Niabella soli | *Niabella soli DSM 19437* | 374374332 |
| Erysipelotrichaceae bacterium GH31 | *Erysipelotrichaceae bacterium 5_2_54FAA* | 293400318 |
| Eubacterium sp. GH31 | *Eubacterium sp. 3_1_31* | 373453378 |
| D. plexippus AG | *Danaus plexippus* | 357610940 |
| B. mori GH31 | *Bombyx mori* | 103058158 |
| C. floridanus AG | *Camponotus floridanus* | 307185295 |
| T. castaneum AG | *Tribolium castaneum* | 91079350 |
| P. humanus corporis AG | *Pediculus humanus corporis* | 242003882 |
| A. carolinensis AG | *Anolis carolinensis* | 327264726 |
| H. sapiens AG | *Homo sapiens* | 31608 |
| D. plexippus HYP | *Danaus plexippus* | 357621138 |
| *C. elegans* HYP | *Caenorhabditis elegans* | 17560798 |
| A. thaliana AG | *Arabidopsis thaliana* | 15237538 |
| H. vulgare AG | *Hordeum vulgare subsp. vulgare* | 208609045 |
| S. cerevisiae GLUC | *Saccharomyces cerevisiae RM11-1a* | 190408622 |
| P. corporis NAG | *Pediculus humanus corporis* | 242019253 |
| C. floridanus NAG | *Camponotus floridanus* | 307188051 |
| H. sapiens NAG | *Homo sapiens* | 119594451 |
| T. castaneum HYP | *Tribolium castaneum* | 189235376 |
| A. carolinensis NAG | *Anolis carolinensis* | 327259569 |
| B. mori HYP | *Bombyx mori* | BGIBMGA012077 |
| D. melanogaster AG | *Drosophila melanogaster* | 21357605 |
| D. plexippus GH31 | *Danaus plexippus* | 357622711 |
| D. melanogaster GH31 | *Drosophila melanogaster* | 24650054 |
| T. castaneum GH31 | *Tribolium castaneum* | 270006118 |
| H. sapiens GH31 | *Homo sapiens* | 153791946 |
| C. floridanus GH31 | *Camponotus floridanus* | 307168057 |
